# A review of fatty acid profiles and antioxidant content in grass-fed and grain-fed beef

**DOI:** 10.1186/1475-2891-9-10

**Published:** 2010-03-10

**Authors:** Cynthia A Daley, Amber Abbott, Patrick S Doyle, Glenn A Nader, Stephanie Larson

**Affiliations:** 1 College of Agriculture, California State University, Chico, CA, USA; 2 University of California Cooperative Extension Service, Davis, CA, USA

## Abstract

Growing consumer interest in grass-fed beef products has raised a number of questions with regard to the perceived differences in nutritional quality between grass-fed and grain-fed cattle. Research spanning three decades suggests that grass-based diets can significantly improve the fatty acid (FA) composition and antioxidant content of beef, albeit with variable impacts on overall palatability. Grass-based diets have been shown to enhance total conjugated linoleic acid (CLA) (C18:2) isomers, *trans *vaccenic acid (TVA) (C18:1 t11), a precursor to CLA, and omega-3 (n-3) FAs on a g/g fat basis. While the overall concentration of total SFAs is not different between feeding regimens, grass-finished beef tends toward a higher proportion of cholesterol neutral stearic FA (C18:0), and less cholesterol-elevating SFAs such as myristic (C14:0) and palmitic (C16:0) FAs. Several studies suggest that grass-based diets elevate precursors for Vitamin A and E, as well as cancer fighting antioxidants such as glutathione (GT) and superoxide dismutase (SOD) activity as compared to grain-fed contemporaries. Fat conscious consumers will also prefer the overall lower fat content of a grass-fed beef product. However, consumers should be aware that the differences in FA content will also give grass-fed beef a distinct grass flavor and unique cooking qualities that should be considered when making the transition from grain-fed beef. In addition, the fat from grass-finished beef may have a yellowish appearance from the elevated carotenoid content (precursor to Vitamin A). It is also noted that grain-fed beef consumers may achieve similar intakes of both n-3 and CLA through the consumption of higher fat grain-fed portions.

## Review Contents

1. Introduction

2. Fatty acid profile in grass-fed beef

3. Impact of grass-finishing on omega-3 fatty acids

4. Impact of grass-finishing on conjugated linoleic acid (CLA) and *trans*-vaccenic acid (TVA)

5. Impact of grass-finishing on β-carotenes/carotenoids

6. Impact of grass-finishing on α-tocopherol

7. Impact of grass-finishing on GT & SOD activity

8. Impact of grass-finishing on flavor and palatability

9. Conclusion

10. References

## Introduction

There is considerable support among the nutritional communities for the diet-heart (lipid) hypothesis, the idea that an imbalance of dietary cholesterol and fats are the primary cause of atherosclerosis and cardiovascular disease (CVD) [1]. Health professionals world-wide recommend a reduction in the overall consumption of SFAs, trans-fatty acids (TAs) and cholesterol, while emphasizing the need to increase intake of n-3 polyunsaturated fats [1,2]. Such broad sweeping nutritional recommendations with regard to fat consumption are largely due to epidemiologic studies showing strong positive correlations between intake of SFA and the incidence of CVD, a condition believed to result from the concomitant rise in serum low-density-lipoprotein (LDL) cholesterol as SFA intake increases [3,4]. For example, it is generally accepted that for every 1% increase in energy from SFA, LDL cholesterol levels reportedly increase by 1.3 to 1.7 mg/dL (0.034 to 0.044 mmol/L) [5-7].

Wide promotion of this correlative data spurred an anti-SFA campaign that reduced consumption of dietary fats, including most animal proteins such as meat, dairy products and eggs over the last 3 decades [8], indicted on their relatively high SFA and cholesterol content. However, more recent lipid research would suggest that not all SFAs have the same impact on serum cholesterol. For instance, lauric acid (C12:0) and myristic acid (C14:0), have a greater total cholesterol raising effect than palmitic acid (C16:0), whereas stearic acid (C18:0) has a neutral effect on the concentration of total serum cholesterol, including no apparent impact on either LDL or HDL. Lauric acid increases total serum cholesterol, although it also decreases the ratio of total cholesterol:HDL because of a preferential increase in HDL cholesterol [5,7,9]. Thus, the individual fatty acid profiles tend to be more instructive than broad lipid classifications with respect to subsequent impacts on serum cholesterol, and should therefore be considered when making dietary recommendations for the prevention of CVD.

Clearly the lipid hypothesis has had broad sweeping impacts; not only on the way we eat, but also on the way food is produced on-farm. Indeed, changes in animal breeding and genetics have resulted in an overall leaner beef product[10]. Preliminary examination of diets containing today's leaner beef has shown a reduction in serum cholesterol, provided that beef consumption is limited to a three ounce portion devoid of all external fat [11]. O'Dea's work was the first of several studies to show today's leaner beef products can reduce plasma LDL concentrations in both normal and hyper-cholesterolemic subjects, theoretically reducing risk of CVD [12-15].

Beyond changes in genetics, some producers have also altered their feeding practices whereby reducing or eliminating grain from the ruminant diet, producing a product referred to as "grass-fed" or "grass-finished". Historically, most of the beef produced until the 1940's was from cattle finished on grass. During the 1950's, considerable research was done to improve the efficiency of beef production, giving birth to the feedlot industry where high energy grains are fed to cattle as means to decrease days on feed and improve marbling (intramuscular fat: IMF). In addition, U.S. consumers have grown accustomed to the taste of grain-fed beef, generally preferring the flavor and overall palatability afforded by the higher energy grain ration[16]. However, changes in consumer demand, coupled with new research on the effect of feed on nutrient content, have a number of producers returning to the pastoral approach to beef production despite the inherent inefficiencies.

Research spanning three decades suggests that grass-only diets can significantly alter the fatty acid composition and improve the overall antioxidant content of beef. It is the intent of this review, to synthesize and summarize the information currently available to substantiate an enhanced nutrient claim for grass-fed beef products as well as to discuss the effects these specific nutrients have on human health.

## Review of fatty acid profiles in grass-fed beef

Red meat, regardless of feeding regimen, is nutrient dense and regarded as an important source of essential amino acids, vitamins A, B_6_, B_12_, D, E, and minerals, including iron, zinc and selenium [17,18]. Along with these important nutrients, meat consumers also ingest a number of fats which are an important source of energy and facilitate the absorption of fat-soluble vitamins including A, D, E and K. According to the ADA, animal fats contribute approximately 60% of the SFA in the American diet, most of which are palmitic acid (C16:0) and stearic acid (C18:0). Stearic acid has been shown to have no net impact on serum cholesterol concentrations in humans[17,19]. In addition, 30% of the FA content in conventionally produced beef is composed of oleic acid (C18:1) [20], a monounsaturated FA (MUFA) that elicits a cholesterol-lowering effect among other healthful attributes including a reduced risk of stroke and a significant decrease in both systolic and diastolic blood pressure in susceptible populations [21].

Be that as it may, changes in finishing diets of conventional cattle can alter the lipid profile in such a way as to improve upon this nutritional package. Although there are genetic, age related and gender differences among the various meat producing species with respect to lipid profiles and ratios, the effect of animal nutrition is quite significant [22]. Regardless of the genetic makeup, gender, age, species or geographic location, direct contrasts between grass and grain rations consistently demonstrate significant differences in the overall fatty acid profile and antioxidant content found in the lipid depots and body tissues [22-24].

Table 1 summarizes the saturated fatty acid analysis for a number of studies whose objectives were to contrast the lipid profiles of cattle fed either a grain or grass diets [25-31]. This table is limited to those studies utilizing the *longissimus dorsi *(loin eye), thereby standardizing the contrasts to similar cuts within the carcass and limits the comparisons to cattle between 20 and 30 months of age. Unfortunately, not all studies report data in similar units of measure (i.e., g/g of fatty acid), so direct comparisons between studies are not possible.

**Table 1:** Comparison of mean saturated fatty acid composition (expressed as mg/g of fatty acid or as a % of total lipid) between grass-fed and grain-fed cattle.

	Fatty Acid						
	
Author, publication year, breed, treatment	C12:0 lauric	C14:0 myristic	C16:0 palmitic	C18:0 stearic	C20:0 arachidic	Total SFA (units as specified)	Total lipid (units as specified)
Alfaia, et al., 2009, Crossbred steers	*g/100 g lipid*						
Grass	0.05	1.24*	18.42*	17.54*	0.25*	38.76	9.76* mg/g muscle
Grain	0.06	1.84*	20.79*	14.96*	0.19*	39.27	13.03* mg/g muscle
Leheska, et al., 2008, Mixed cattle	*g/100 g lipid*						
Grass	0.05	2.84*	26.9	17.0*	0.13*	48.8*	2.8* % of muscle
Grain	0.07	3.45*	26.3	13.2*	0.08*	45.1*	4.4* % of muscle
Garcia et al., 2008, Angus X-bred steers	*% of total FA*						
Grass	na	2.19	23.1	13.1*	na	38.4*	2.86* %IMF
Grain	na	2.44	22.1	10.8*	na	35.3*	3.85* %IMF
Ponnampalam, et al., 2006, Angus steers	*mg/100 g muscle tissue*						
Grass	na	56.9*	508*	272.8	na	900*	2.12%* % of muscle
Grain	na	103.7*	899*	463.3	na	1568*	3.61%* % of muscle
Nuernberg, et al., 2005, Simmental bulls	*% of total intramuscular fat reported as LSM*						
Grass	0.04	1.82	22.56*	17.64*	na	43.91	1.51* % of muscle
Grain	0.05	1.96	24.26*	16.80*	na	44.49	2.61* % of muscle
Descalzo, et al., 2005 Crossbred Steers	*% of total FA*						
Grass	na	2.2	22.0	19.1	na	42.8	2.7* %IMF
Grain	na	2.0	25.0	18.2	na	45.5	4.7* %IMF
Realini, et al., 2004, Hereford steers	*% fatty acid within intramuscular fat*						
Grass	na	1.64*	21.61*	17.74*	na	49.08	1.68* % of muscle
Grain	na	2.17*	24.26*	15.77*	na	47.62	3.18* % of muscle

*Indicates a significant difference (at least P < 0.05) between feeding regimens was reported within each respective study. "na" indicates that the value was not reported in the original study.

Table 1 reports that grass finished cattle are typically lower in total fat as compared to grain-fed contemporaries. Interestingly, there is no consistent difference in total SFA content between these two feeding regimens. Those SFA's considered to be more detrimental to serum cholesterol levels, i.e., myristic (C14:0) and palmitic (C16:0), were higher in grain-fed beef as compared to grass-fed contemporaries in 60% of the studies reviewed. Grass finished meat contains elevated concentrations of stearic acid (C18:0), the only saturated fatty acid with a net neutral impact on serum cholesterol. Thus, grass finished beef tends to produce a more favorable SFA composition although little is known of how grass-finished beef would ultimately impact serum cholesterol levels in hyper-cholesterolemic patients as compared to a grain-fed beef.

Like SFA intake, dietary cholesterol consumption has also become an important issue to consumers. Interestingly, beef's cholesterol content is similar to other meats (beef 73; pork 79; lamb 85; chicken 76; and turkey 83 mg/100 g) [32], and can therefore be used interchangeably with white meats to reduce serum cholesterol levels in hyper-cholesterolemic individuals[11,33]. Studies have shown that breed, nutrition and sex do not affect the cholesterol concentration of bovine skeletal muscle, rather cholesterol content is highly correlated to IMF concentrations[34]. As IMF levels rise, so goes cholesterol concentrations per gram of tissue [35]. Because pasture raised beef is lower in overall fat [24-27,30], particularly with respect to marbling or IMF [26,36], it would seem to follow that grass-finished beef would be lower in overall cholesterol content although the data is very limited. Garcia et al (2008) report 40.3 and 45.8 grams of cholesterol/100 grams of tissue in pastured and grain-fed steers, respectively (P < 0.001) [24].

Interestingly, grain-fed beef consistently produces higher concentrations of MUFAs as compared to grass-fed beef, which include FAs such as oleic acid (C18:1 cis-9), the primary MUFA in beef. A number of epidemiological studies comparing disease rates in different countries have suggested an inverse association between MUFA intake and mortality rates to CVD [3,21]. Even so, grass-fed beef provides a higher concentration of TVA (C18:1 *t*11), an important MUFA for de novo synthesis of conjugated linoleic acid (CLA: C18:2 *c*-9, *t*-11), a potent anti-carcinogen that is synthesized within the body tissues [37]. Specific information relative to the health benefits of CLA and its biochemistry will be detailed later.

The important polyunsaturated fatty acids (PUFAs) in conventional beef are linoleic acid (C18:2), alpha-linolenic acid (C18:3), described as the essential FAs, and the long-chain fatty acids including arachidonic acid (C20:4), eicosapentaenoic acid (C20:5), docosanpetaenoic acid (C22:5) and docosahexaenoic acid (C22:6) [38]. The significance of nutrition on fatty acid composition is clearly demonstrated when profiles are examined by omega 6 (n-6) and omega 3 (n-3) families. Table 2 shows no significant change to the overall concentration of n-6 FAs between feeding regimens, although grass-fed beef consistently shows a higher concentrations of n-3 FAs as compared to grain-fed contemporaries, creating a more favorable n-6:n-3 ratio. There are a number of studies that report positive effects of improved n-3 intake on CVD and other health related issues discussed in more detail in the next section.

**Table 2:** Comparison of mean polyunsatured fatty acid composition (expressed as mg/g of fatty acid or as a % of total lipid) between grass-fed and grain-fed cattle.

	Fatty Acid											
	
Author, publication year, breed, treatment	C18:1 t11 Vaccenic Acid	C18:2 n-6 Linoleic	Total CLA	C18:3 n-3 Linolenic	C20:5n-3 EPA	C22:5n-3 DPA	C22:6n-3 DHA	Total PUFA	Total MUFA	Total n-6	Total n-3	n-6/n-3 ratio
Alfaia, et al., 2009, Crossbred steers	*g/100 g lipid*											
Grass	1.35	12.55	5.14*	5.53*	2.13*	2.56*	0.20*	28.99*	24.69*	17.97*	10.41*	1.77*
Grain	0.92	11.95	2.65*	0.48*	0.47*	0.91*	0.11*	19.06*	34.99*	17.08	1.97*	8.99*
Leheska, et al., 2008, Mixed cattle	*g/100 g lipid*											
Grass	2.95*	2.01	0.85*	0.71*	0.31	0.24*	na	3.41	42.5*	2.30	1.07*	2.78*
Grain	0.51*	2.38	0.48*	0.13*	0.19	0.06*	na	2.77	46.2*	2.58	0.19*	13.6*
Garcia, et al., 2008, Angus steers	*% of total FAs*											
Grass	3.22*	3.41	0.72*	1.30*	0.52*	0.70*	0.43*	7.95	37.7*	5.00*	2.95*	1.72*
Grain	2.25*	3.93	0.58*	0.74*	0.12*	0.30*	0.14*	9.31	40.8*	8.05*	0.86*	10.38*
Ponnampalam, et al., 2006, Angus steers	*mg/100 g muscle tissue*											
Grass	na	108.8*	14.3	32.4*	24.5*	36.5*	4.2	na	930*	191.6	97.6*	1.96*
Grain	na	167.4*	16.1	14.9*	13.1*	31.6*	3.7	na	1729*	253.8	63.3*	3.57*
Nuernberg, et al., 2005, Simmental bulls	*% of total fatty acids*											
Grass	na	6.56	0.87*	2.22*	0.94*	1.32*	0.17*	14.29*	56.09	9.80	4.70*	2.04*
Grain	na	5.22	0.72*	0.46*	0.08*	0.29*	0.05*	9.07*	55.51	7.73	0.90*	8.34*
Descalzo, et al., 2005, Crossbred steers	*% of total FAs*											
Grass	4.2*	5.4	na	1.4*	tr	0.6	tr	10.31*	34.17*	7.4	2.0	3.72*
Grain	2.8*	4.7	na	0.7*	tr	0.4	tr	7.29*	37.83*	6.3	1.1	5.73*
Realini, et al., 2004, Hereford steers	*% fatty acid within intramuscular fat*											
Grass	na	3.29*	0.53*	1.34*	0.69*	1.04*	0.09	9.96*	40.96*	na	na	1.44*
Grain	na	2.84*	0.25*	0.35*	0.30*	0.56*	0.09	6.02*	46.36*	na	na	3.00*

* Indicates a significant difference (at least P < 0.05) between feeding regimens within each respective study reported. "na" indicates that the value was not reported in the original study. "tr" indicates trace amounts detected.

## Review of Omega-3: Omega-6 fatty acid content in grass-fed beef

**Figure 1:**
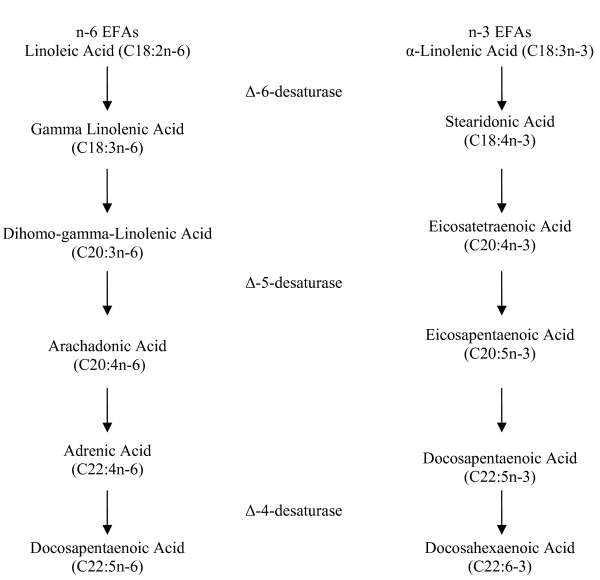
**Linoleic (C18:2n-6) and α-Linolenic (C18:3n-3) Acid metabolism and elongation**. (Adapted from *Simopoulos et al., 1991*)

There are two essential fatty acids (EFAs) in human nutrition: α-linolenic acid (αLA), an omega-3 fatty acid; and linoleic acid (LA), an omega-6 fatty acid. The human body cannot synthesize essential fatty acids, yet they are critical to human health; for this reason, EFAs must be obtained from food. Both αLA and LA are polyunsaturated and serve as precursors of other important compounds. For instance, αLA is the precursor for the omega-3 pathway. Likewise, LA is the parent fatty acid in the omega-6 pathway. Omega-3 (n-3) and omega-6 (n-6) fatty acids are two separate distinct families, yet they are synthesized by some of the same enzymes; specifically, delta-5-desaturase and delta-6-desaturase. Excess of one family of FAs can interfere with the metabolism of the other, reducing its incorporation into tissue lipids and altering their overall biological effects [39]. Figure 1 depicts a schematic of n-6 and n-3 metabolism and elongation within the body [40].

A healthy diet should consist of roughly one to four times more omega-6 fatty acids than omega-3 fatty acids. The typical American diet tends to contain 11 to 30 times more omega -6 fatty acids than omega -3, a phenomenon that has been hypothesized as a significant factor in the rising rate of inflammatory disorders in the United States[40]. Table 2 shows significant differences in n-6:n-3 ratios between grass-fed and grain-fed beef, with and overall average of 1.53 and 7.65 for grass-fed and grain-fed, respectively, for all studies reported in this review.

The major types of omega-3 fatty acids used by the body include: α-linolenic acid (C18:3n-3, αLA), eicosapentaenoic acid (C20:5n-3, EPA), docosapentaenoic acid (C22:5n-3, DPA), and docosahexaenoic acid (C22:6n-3, DHA). Once eaten, the body converts αLA to EPA, DPA and DHA, albeit at low efficiency. Studies generally agree that whole body conversion of αLA to DHA is below 5% in humans, the majority of these long-chain FAs are consumed in the diet [41].

The omega-3 fatty acids were first discovered in the early 1970's when Danish physicians observed that Greenland Eskimos had an exceptionally low incidence of heart disease and arthritis despite the fact that they consumed a diet high in fat. These early studies established fish as a rich source of n-3 fatty acids. More recent research has established that EPA and DHA play a crucial role in the prevention of atherosclerosis, heart attack, depression and cancer [40,42]. In addition, omega-3 consumption reduced the inflammation caused by rheumatoid arthritis [43,44].

The human brain has a high requirement for DHA; low DHA levels have been linked to low brain serotonin levels, which are connected to an increased tendency for depression and suicide. Several studies have established a correlation between low levels of omega -3 fatty acids and depression. High consumption of omega-3 FAs is typically associated with a lower incidence of depression, a decreased prevalence of age-related memory loss and a lower risk of developing Alzheimer's disease [45-51].

The National Institutes of Health has published recommended daily intakes of FAs; specific recommendations include 650 mg of EPA and DHA, 2.22 g/day of αLA and 4.44 g/day of LA. However, the Institute of Medicine has recommended DRI (dietary reference intake) for LA (omega-6) at 12 to 17 g and αLA (omega-3) at 1.1 to 1.6 g for adult women and men, respectively. Although seafood is the major dietary source of n-3 fatty acids, a recent fatty acid intake survey indicated that red meat also serves as a significant source of n-3 fatty acids for some populations [52].

Sinclair and co-workers were the first to show that beef consumption increased serum concentrations of a number of n-3 fatty acids including, EPA, DPA and DHA in humans [40]. Likewise, there are a number of studies that have been conducted with livestock which report similar findings, i.e., animals that consume rations high in precursor lipids produce a meat product higher in the essential fatty acids [53,54]. For instance, cattle fed primarily grass significantly increased the omega-3 content of the meat and also produced a more favorable omega-6 to omega-3 ratio than grain-fed beef [46,55-57].

Table 2 shows the effect of ration on polyunsaturated fatty acid composition from a number of recent studies that contrast grass-based rations to conventional grain feeding regimens [24-28,30,31]. Grass-based diets resulted in significantly higher levels of omega-3 within the lipid fraction of the meat, while omega-6 levels were left unchanged. In fact, as the concentration of grain is increased in the grass-based diet, the concentration of n-3 FAs decreases in a linear fashion. Grass-finished beef consistently produces a higher concentration of n-3 FAs (without effecting n-6 FA content), resulting in a more favorable n-6:n-3 ratio.

The amount of total lipid (fat) found in a serving of meat is highly dependent upon the feeding regimen as demonstrated in Tables 1 and 2. Fat will also vary by cut, as not all locations of the carcass will deposit fat to the same degree. Genetics also play a role in lipid metabolism creating significant breed effects. Even so, the effect of feeding regimen is a very powerful determinant of fatty acid composition.

## Review of conjugated linoleic acid (CLA) and *trans *vaccenic acid (TVA) in grass-fed beef

Conjugated linoleic acids make up a group of polyunsaturated FAs found in meat and milk from ruminant animals and exist as a general mixture of conjugated isomers of LA. Of the many isomers identified, the *cis*-9, *trans*-11 CLA isomer (also referred to as rumenic acid or RA) accounts for up to 80-90% of the total CLA in ruminant products [58]. Naturally occurring CLAs originate from two sources: bacterial isomerization and/or biohydrogenation of polyunsaturated fatty acids (PUFA) in the rumen and the desaturation of trans-fatty acids in the adipose tissue and mammary gland [59,60].

**Figure 2:**
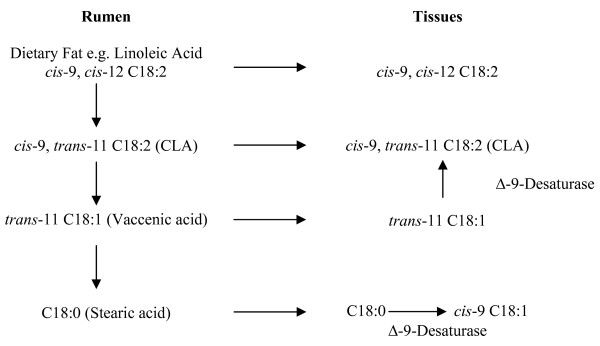
**De novo synthesis of CLA from 11t-C18:1 vaccenic acid**. (Adapted from *Bauman et al., 1999*)

Microbial biohydrogenation of LA and αLA by an anaerobic rumen bacterium *Butyrivibrio fibrisolvens *is highly dependent on rumen pH [61]. Grain consumption decreases rumen pH, reducing *B. fibrisolven *activity, conversely grass-based diets provide for a more favorable rumen environment for subsequent bacterial synthesis [62]. Rumen pH may help to explain the apparent differences in CLA content between grain and grass-finished meat products (see Table 2). De novo synthesis of CLA from 11*t*-C18:1 TVA has been documented in rodents, dairy cows and humans. Studies suggest a linear increase in CLA synthesis as the TVA content of the diet increased in human subjects [63]. The rate of conversion of TVA to CLA has been estimated to range from 5 to 12% in rodents to 19 to 30% in humans[64]. True dietary intake of CLA should therefore consider native 9*c*11*t*-C18:2 (actual CLA) as well as the 11*t*-C18:1 (potential CLA) content of foods [65,66]. Figure 2 portrays de novo synthesis pathways of CLA from TVA [37].

Natural augmentation of CLA *c*9*t*11 and TVA within the lipid fraction of beef products can be accomplished through diets rich in grass and lush green forages. While precursors can be found in both grains and lush green forages, grass-fed ruminant species have been shown to produce 2 to 3 times more CLA than ruminants fed in confinement on high grain diets, largely due to a more favorable rumen pH [34,56,57,67] (see Table 2).

The impact of feeding practices becomes even more evident in light of recent reports from Canada which suggests a shift in the predominate *trans *C18:1 isomer in grain-fed beef. Dugan et al (2007) reported that the major *trans *isomer in beef produced from a 73% barley grain diet is 10*t*-18:1 (2.13% of total lipid) rather than 11*t*-18:1 (TVA) (0.77% of total lipid), a finding that is not particularly favorable considering the data that would support a negative impact of 10*t*-18:1 on LDL cholesterol and CVD [68,69].

Over the past two decades numerous studies have shown significant health benefits attributable to the actions of CLA, as demonstrated by experimental animal models, including actions to reduce carcinogenesis, atherosclerosis, and onset of diabetes [70-72]. Conjugated linoleic acid has also been reported to modulate body composition by reducing the accumulation of adipose tissue in a variety of species including mice, rats, pigs, and now humans [73-76]. These changes in body composition occur at ultra high doses of CLA, dosages that can only be attained through synthetic supplementation that may also produce ill side-effects, such as gastrointestinal upset, adverse changes to glucose/insulin metabolism and compromised liver function [77-81]. A number of excellent reviews on CLA and human health can be found in the literature [61,82-84].

Optimal dietary intake remains to be established for CLA. It has been hypothesized that 95 mg CLA/day is enough to show positive effects in the reduction of breast cancer in women utilizing epidemiological data linking increased milk consumption with reduced breast cancer[85]. Ha et al. (1989) published a much more conservative estimate stating that 3 g/day CLA is required to promote human health benefits[86]. Ritzenthaler et al. (2001) estimated CLA intakes of 620 mg/day for men and 441 mg/day for women are necessary for cancer prevention[87]. Obviously, all these values represent rough estimates and are mainly based on extrapolated animal data. What is clear is that we as a population do not consume enough CLA in our diets to have a significant impact on cancer prevention or suppression. Reports indicate that Americans consume between 150 to 200 mg/day, Germans consumer slightly more between 300 to 400 mg/day[87], and the Australians seem to be closer to the optimum concentration at 500 to 1000 mg/day according to Parodi (1994) [88].

## Review of pro-Vitamin A/β-carotene in grass-fed meat

Carotenoids are a family of compounds that are synthesized by higher plants as natural plant pigments. Xanthophylls, carotene and lycopene are responsible for yellow, orange and red coloring, respectively. Ruminants on high forage rations pass a portion of the ingested carotenoids into the milk and body fat in a manner that has yet to be fully elucidated. Cattle produced under extensive grass-based production systems generally have carcass fat which is more yellow than their concentrate-fed counterparts caused by carotenoids from the lush green forages. Although yellow carcass fat is negatively regarded in many countries around the world, it is also associated with a healthier fatty acid profile and a higher antioxidant content [89].

Plant species, harvest methods, and season, all have significant impacts on the carotenoid content of forage. In the process of making silage, haylage or hay, as much as 80% of the carotenoid content is destroyed [90]. Further, significant seasonal shifts occur in carotenoid content owing to the seasonal nature of plant growth.

Carotenes (mainly β-carotene) are precursors of retinol (Vitamin A), a critical fat-soluble vitamin that is important for normal vision, bone growth, reproduction, cell division, and cell differentiation [91]. Specifically, it is responsible for maintaining the surface lining of the eyes and also the lining of the respiratory, urinary, and intestinal tracts. The overall integrity of skin and mucous membranes is maintained by vitamin A, creating a barrier to bacterial and viral infection [15,92]. In addition, vitamin A is involved in the regulation of immune function by supporting the production and function of white blood cells [12,13].

The current recommended intake of vitamin A is 3,000 to 5,000 IU for men and 2,300 to 4,000 IU for women [93], respectively, which is equivalent to 900 to 1500 μg (micrograms) (Note: DRI as reported by the Institute of Medicine for non-pregnant/non-lactating adult females is 700 μg/day and males is 900 μg/day or 2,300 - 3,000 I U (assuming conversion of 3.33 IU/μg). While there is no RDA (Required Daily Allowance) for β-carotene or other pro-vitamin A carotenoids, the Institute of Medicine suggests consuming 3 mg of β-carotene daily to maintain plasma β-carotene in the range associated with normal function and a lowered risk of chronic diseases (NIH: Office of Dietary Supplements).

The effects of grass feeding on beta-carotene content of beef was described by Descalzo et al. (2005) who found pasture-fed steers incorporated significantly higher amounts of beta-carotene into muscle tissues as compared to grain-fed animals [94]. Concentrations were 0.45 μg/g and 0.06 μg/g for beef from pasture and grain-fed cattle respectively, demonstrating a 7 fold increase in β-carotene levels for grass-fed beef over the grain-fed contemporaries. Similar data has been reported previously, presumably due to the high β-carotene content of fresh grasses as compared to cereal grains[38,55,95-97]. (see Table 3)

**Table 3:** Comparison of mean β-carotene vitamin content in fresh beef from grass-fed and grain-fed cattle.

	β-carotene	
	
Author, year, animal class	Grass-fed (ug/g tissue)	Grain-fed (ug/g tissue)
Insani et al., 2007, Crossbred steers	0.74*	0.17*
Descalzo et al., 2005 Crossbred steers	0.45*	0.06*
Yang et al., 2002, Crossbred steers	0.16*	0.01*

* Indicates a significant difference (at least P < 0.05) between feeding regimens was reported within each respective study.

## Review of Vitamin E/α-tocopherol in grass-fed beef

Vitamin E is also a fat-soluble vitamin that exists in eight different isoforms with powerful antioxidant activity, the most active being α-tocopherol [98]. Numerous studies have shown that cattle finished on pasture produce higher levels of α-tocopherol in the final meat product than cattle fed high concentrate diets[23,28,94,97,99-101] (see Table 4).

**Table 4:** Comparison of mean α-tocopherol vitamin content in fresh beef from grass-fed and grain-fed cattle.

	*α-tocopherol*	
	
Author, year, animal class	Grass-fed (ug/g tissue)	Grain-fed (ug/g tissue)
De la Fuente et al., 2009, Mixed cattle	4.07*	0.75*
Descalzo, et al., 2008, Crossbred steers	3.08*	1.50*
Insani et al., 2007, Crossbred steers	2.1*	0.8*
Descalzo, et al., 2005, Crosbred steers	4.6*	2.2*
Realini et al., 2004, Hereford steers	3.91*	2.92*
Yang et al., 2002, Crossbred steers	4.5*	1.8*

* Indicates a significant difference (at least P < 0.05) between feeding regimens was reported within each respective study.

Antioxidants such as vitamin E protect cells against the effects of free radicals. Free radicals are potentially damaging by-products of metabolism that may contribute to the development of chronic diseases such as cancer and cardiovascular disease.

Preliminary research shows vitamin E supplementation may help prevent or delay coronary heart disease [102-105]. Vitamin E may also block the formation of nitrosamines, which are carcinogens formed in the stomach from nitrates consumed in the diet. It may also protect against the development of cancers by enhancing immune function [106]. In addition to the cancer fighting effects, there are some observational studies that found lens clarity (a diagnostic tool for cataracts) was better in patients who regularly used vitamin E [107,108]. The current recommended intake of vitamin E is 22 IU (natural source) or 33 IU (synthetic source) for men and women [93,109], respectively, which is equivalent to 15 milligrams by weight.

The concentration of natural α-tocopherol (vitamin E) found in grain-fed beef ranged between 0.75 to 2.92 μg/g of muscle whereas pasture-fed beef ranges from 2.1 to 7.73 μg/g of tissue depending on the type of forage made available to the animals (Table 4). Grass finishing increases α-tocopherol levels three-fold over grain-fed beef and places grass-fed beef well within range of the muscle α-tocopherol levels needed to extend the shelf-life of retail beef (3 to 4 μg α-tocopherol/gram tissue) [110]. Vitamin E (α-tocopherol) acts post-mortem to delay oxidative deterioration of the meat; a process by which myoglobin is converted into brown metmyoglobin, producing a darkened, brown appearance to the meat. In a study where grass-fed and grain-fed beef were directly compared, the bright red color associated with oxymyoglobin was retained longer in the retail display in the grass-fed group, even thought the grass-fed meat contains a higher concentration of more oxidizable n-3 PUFA. The authors concluded that the antioxidants in grass probably caused higher tissue levels of vitamin E in grazed animals with benefits of lower lipid oxidation and better color retention despite the greater potential for lipid oxidation[111].

## Review of antioxidant enzyme content in grass-fed beef

Glutathione (GT), is a relatively new protein identified in foods. It is a tripeptide composed of cysteine, glutamic acid and glycine and functions as an antioxidant primarily as a component of the enzyme system containing GT oxidase and reductase. Within the cell, GT has the capability of quenching free radicals (like hydrogen peroxide), thus protecting the cell from oxidized lipids or proteins and prevent damage to DNA. GT and its associated enzymes are found in virtually all plant and animal tissue and is readily absorbed in the small intestine[112].

Although our knowledge of GT content in foods is still somewhat limited, dairy products, eggs, apples, beans, and rice contain very little GT (< 3.3 mg/100 g). In contrast, fresh vegetables (e.g., asparagus 28.3 mg/100 g) and freshly cooked meats, such as ham and beef (23.3 mg/100 g and 17.5 mg/100 g, respectively), are high in GT [113].

Because GT compounds are elevated in lush green forages, grass-fed beef is particularly high in GT as compared to grain-fed contemporaries. Descalzo et al. (2007) reported a significant increase in GT molar concentrations in grass-fed beef [114]. In addition, grass-fed samples were also higher in superoxide dismutase (SOD) and catalase (CAT) activity than beef from grain-fed animals[115]. Superoxide dismutase and catalase are coupled enzymes that work together as powerful antioxidants, SOD scavenges superoxide anions by forming hydrogen peroxide and CAT then decomposes the hydrogen peroxide to H_2_O and O_2_. Grass only diets improve the oxidative enzyme concentration in beef, protecting the muscle lipids against oxidation as well as providing the beef consumer with an additional source of antioxidant compounds.

## Issues related to flavor and palatability of grass-fed beef

Maintaining the more favorable lipid profile in grass-fed beef requires a high percentage of lush fresh forage or grass in the ration. The higher the concentration of fresh green forages, the higher the αLA precursor that will be available for CLA and n-3 synthesis [53,54]. Fresh pasture forages have 10 to 12 times more C18:3 than cereal grains [116]. Dried or cured forages, such as hay, will have a slightly lower amount of precursor for CLA and n-3 synthesis. Shifting diets to cereal grains will cause a significant change in the FA profile and antioxidant content within 30 days of transition [57].

Because grass-finishing alters the biochemistry of the beef, aroma and flavor will also be affected. These attributes are directly linked to the chemical makeup of the final product. In a study comparing the flavor compounds between cooked grass-fed and grain-fed beef, the grass-fed beef contained higher concentrations of diterpenoids, derivatives of chlorophyll call phyt-1-ene and phyt-2-ene, that changed both the flavor and aroma of the cooked product [117]. Others have identified a "green" odor from cooked grass-fed meat associated with hexanals derived from oleic and αLA FAs. In contrast to the "green" aroma, grain-fed beef was described as possessing a "soapy" aroma, presumably from the octanals formed from LA that is found in high concentration in grains [118]. Grass-fed beef consumers can expect a different flavor and aroma to their steaks as they cook on the grill. Likewise, because of the lower lipid content and high concentration of PUFAs, cooking time will be reduced. For an exhaustive look at the effect of meat compounds on flavor, see Calkins and Hodgen (2007) [119].

With respect to palatability, grass-fed beef has historically been less well accepted in markets where grain-fed products predominant. For example, in a study where British lambs fed grass and Spanish lambs fed milk and concentrates were assessed by British and Spanish taste panels, both found the British lamb to have a higher odor and flavor intensity. However, the British panel preferred the flavor and overall eating quality of the grass-fed lamb, the Spanish panel much preferred the Spanish fed lamb [120]. Likewise, the U.S. is well known for producing corn-fed beef, taste panels and consumers who are more familiar with the taste of corn-fed beef seem to prefer it as well [16]. An individual usually comes to prefer the foods they grew up eating, making consumer sensory panels more of an art than science [36]. Trained taste panels, i.e., persons specifically trained to evaluate sensory characteristics in beef, found grass-fed beef less palatable than grain-fed beef in flavor and tenderness [119,121].

## Conclusion

Research spanning three decades supports the argument that grass-fed beef (on a g/g fat basis), has a more desirable SFA lipid profile (more C18:0 cholesterol neutral SFA and less C14:0 & C16:0 cholesterol elevating SFAs) as compared to grain-fed beef. Grass-finished beef is also higher in total CLA (C18:2) isomers, TVA (C18:1 t11) and n-3 FAs on a g/g fat basis. This results in a better n-6:n-3 ratio that is preferred by the nutritional community. Grass-fed beef is also higher in precursors for Vitamin A and E and cancer fighting antioxidants such as GT and SOD activity as compared to grain-fed contemporaries.

Grass-fed beef tends to be lower in overall fat content, an important consideration for those consumers interested in decreasing overall fat consumption. Because of these differences in FA content, grass-fed beef also possesses a distinct grass flavor and unique cooking qualities that should be considered when making the transition from grain-fed beef. To maximize the favorable lipid profile and to guarantee the elevated antioxidant content, animals should be finished on 100% grass or pasture-based diets.

Grain-fed beef consumers may achieve similar intakes of both n-3 and CLA through consumption of higher fat portions with higher overall palatability scores. A number of clinical studies have shown that today's lean beef, regardless of feeding strategy, can be used interchangeably with fish or skinless chicken to reduce serum cholesterol levels in hypercholesterolemic patients.

## Abbreviations

c: cis; t: trans; FA: fatty acid; SFA: saturated fatty acid; PUFA: polyunsaturated fatty acid; MUFA: monounsaturated fatty acid; CLA: conjugated linoleic acid; TVA: trans-vaccenic acid; EPA: eicosapentaenoic acid; DPA: docosapentaenoic acid; DHA: docosahexaenoic acid; GT: glutathione; SOD: superoxide dismutase; CAT: catalase.

## Competing interests

The authors declare that they have no competing interests.

## Authors' contributions

CAD was responsible for the literature review, completed most of the primary writing, created the manuscript and worked through the submission process; AA conducted the literature search, organized the articles according to category, completed some of the primary writing and served as editor; SPD conducted a portion of the literature review and served as editor for the manuscript; GAN conducted a portion of the literature review and served as editor for the manuscript; SL conducted a portion o the literature review and served as editor for the manuscript. All authors read and approved the final manuscript.
